# Supplementary material for: A Single Dose of AC102 Reverts Tinnitus by Restoring Ribbon Synapses in Noise-Exposed Mongolian Gerbils
Source: Int J Mol Sci. 2025 May 27;26(11):5124. doi: 10.3390/ijms26115124 (PMC12154400; doi:10.3390/ijms26115124)
Supplement: Supplementary file 1 [file ijms-26-05124-s001.zip › ijms-3589059-supplementary.pdf]

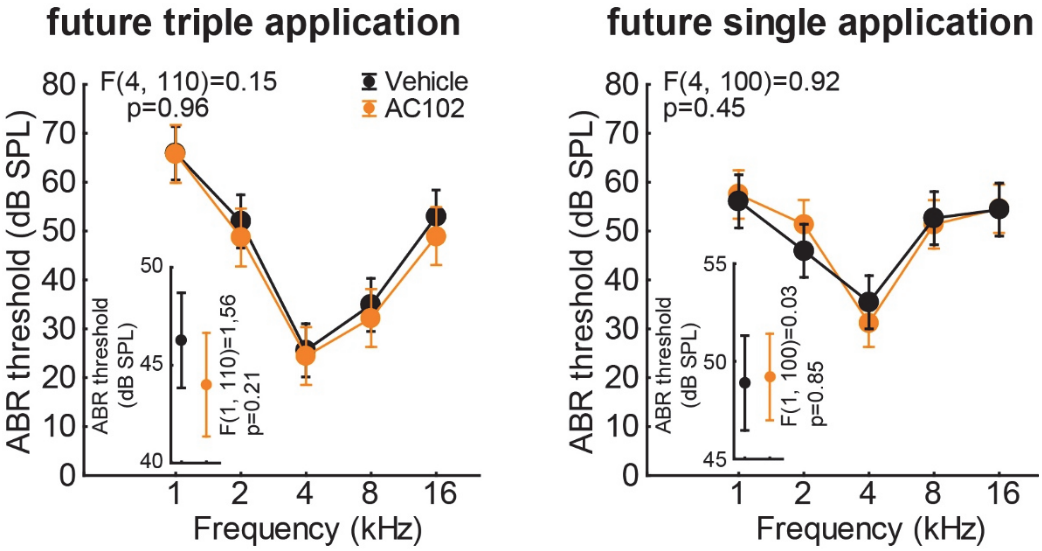

Supplementary Figure S1: Interaction plot of 2-factorial ANOVAs (*future treatment* and *frequency*) of healthy animals' mean ABR thresholds (± 95% CI) in both experimental conditions. Insets show factor *future treatment*.

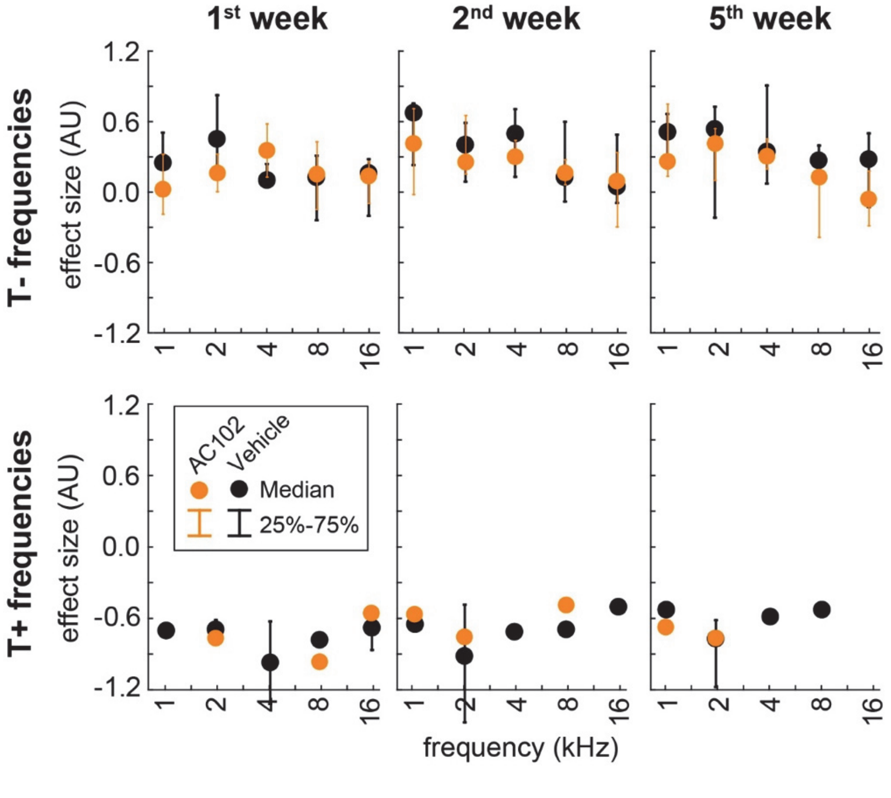

Supplementary Figure S2: Median and interquartile range of the GPIAS response effect size over time in the triple application protocol. The upper panels show the effect sizes of animals that did not develop tinnitus over time (no tinnitus, T-). Data for animals that scored tinnitus positive (tinnitus, T+) are presented in the lower panels. Values are relative to baseline prior to noise exposure.

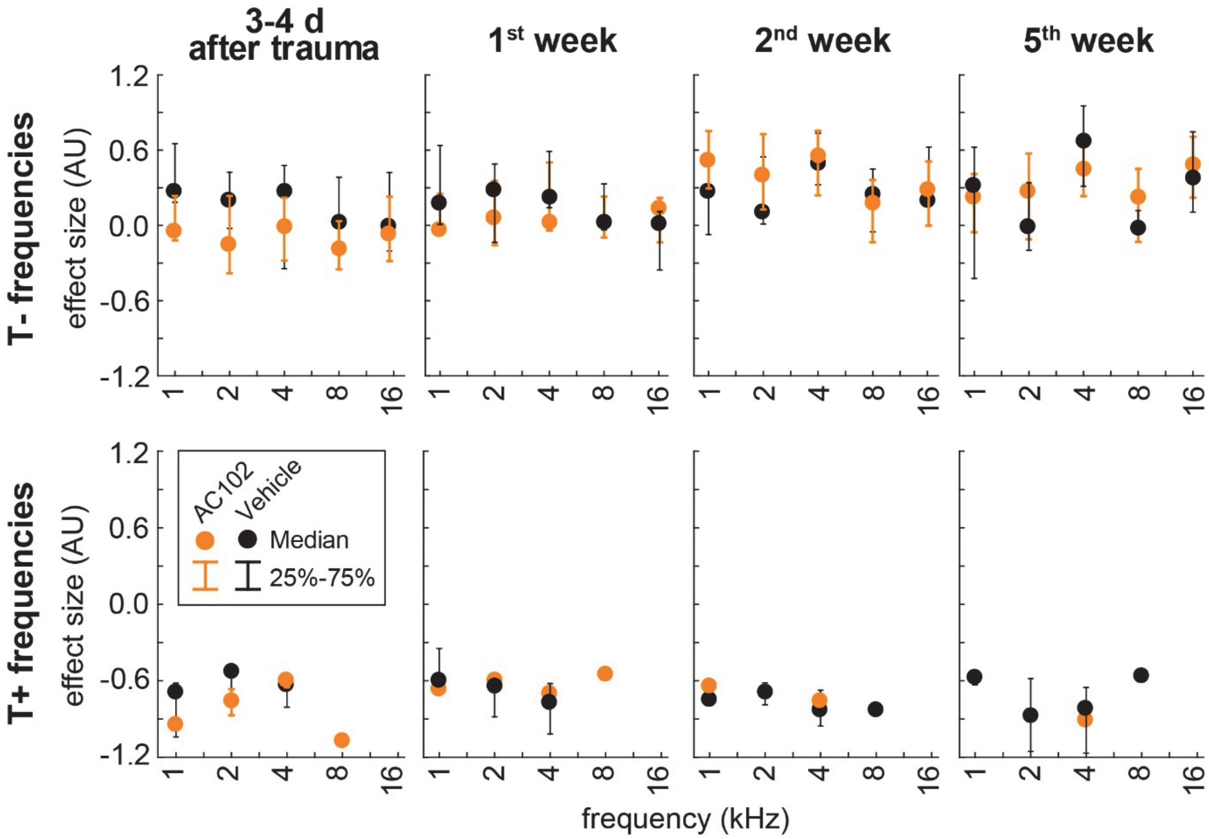

Supplementary Figure S3: Median and interquartile range of the GPIAS response effect size over time in the single application protocol. The upper panels show the effect sizes of animals that did not develop tinnitus over time (no tinnitus, T-). Data for animals that scored tinnitus positive (tinnitus, T+) are presented in the lower panels. Values are relative to baseline prior to noise exposure.
